# Quantification of Epileptogenic Network From Stereo EEG Recordings Using Epileptogenicity Ranking Method

**DOI:** 10.3389/fneur.2021.738111

**Published:** 2021-11-03

**Authors:** Harilal Parasuram, Siby Gopinath, Ashok Pillai, Shyam Diwakar, Anand Kumar

**Affiliations:** ^1^Amrita Advanced Centre for Epilepsy (AACE), Amrita Institute of Medical Sciences, Amrita Vishwa Vidyapeetham, Kochi, India; ^2^Department of Neurology, Amrita Institute of Medical Sciences, Amrita Vishwa Vidyapeetham, Kochi, India; ^3^Amrita Mind Brain Center, Amrita Vishwa Vidyapeetham, Kollam, India; ^4^Department of Neurosurgery, Amrita Institute of Medical Sciences, Amrita Vishwa Vidyapeetham, Kochi, India

**Keywords:** epileptogenicity, SEEG, epileptogenicity rank, intracranial EEG (iEEG), stereo EEG

## Abstract

**Introduction:** Precise localization of the epileptogenic zone is very essential for the success of epilepsy surgery. Epileptogenicity index (EI) computationally estimates epileptogenicity of brain structures based on the temporal domain parameters and magnitude of ictal discharges. This method works well in cases of mesial temporal lobe epilepsy but it showed reduced accuracy in neocortical epilepsy. To overcome this scenario, in this study, we propose Epileptogenicity Rank (ER), a modified method of EI for quantifying epileptogenicity, that is based on spatio-temporal properties of Stereo EEG (SEEG).

**Methods:** Energy ratio during ictal discharges, the time of involvement and Euclidean distance between brain structures were used to compute the ER. Retrospectively, we localized the EZ for 33 patients (9 for mesial-temporal lobe epilepsy and 24 for neocortical epilepsy) using post op MRI and Engel 1 surgical outcome at a mean of 40.9 months and then optimized the ER in this group.

**Results:** Epileptic network estimation based on ER successfully differentiated brain regions involved in the seizure onset from the propagation network. ER was calculated at multiple thresholds leading to an optimum value that differentiated the seizure onset from the propagation network. We observed that ER < 7.1 could localize the EZ in neocortical epilepsy with a sensitivity of 94.6% and specificity of 98.3% and ER < 7.3 in mesial temporal lobe epilepsy with a sensitivity of 95% and specificity of 98%. In non-seizure-free patients, the EZ localization based on ER pointed to brain area beyond the cortical resections.

**Significance:** Methods like ER can improve the accuracy of EZ localization for brain resection and increase the precision of minimally invasive surgery techniques (radio-frequency or laser ablation) by identifying the epileptic hubs where the lesion is extensive or in nonlesional cases. For inclusivity with other clinical applications, this ER method has to be studied in more patients.

## Highlights

- Epileptogenicity Rank (ER) is a modified method of Epileptogenicity Index (EI) for quantifying epileptogenicity of brain structures in epilepsy patients.
- The ER method employs temporal and spatial properties of intracranial Stereo EEG (SEEG) to localize the epileptogenic zone.
- In neocortical epilepsy, the ER method has higher EZ localization accuracy than the epileptogenicity index method.

## Introduction

According to Lüders, the epileptogenic zone (EZ) is the minimum amount of brain area that requires to be resected to render the patient seizure-free (1). It is approximated by the various zones, with the irritative zone almost always significantly larger than the EZ (2). Precise localization of the EZ is always the major challenge in epilepsy surgery (3). The presurgical workup involves various evaluations including EEG, MRI, PET, SPECT, ESI and MEG that study the electrical, structural and functional abnormalities in the patient brain (4). The recommendation from presurgical evaluation brings a possible hypothesis about the seizure onset and propagation zone. To prove this hypothesis of epileptogenic zone, especially in non-lesional, multilesional or other difficult cases, intracranial stereo EEG (SEEG) electrodes are guided to the suspected brain areas to record the local brain activity during interictal to ictal transition (5–7). SEEGs are reviewed at various intervals, including interictal, preictal, and interictal to ictal transition states to localize the EZ. The visual analysis of SEEG during interictal to ictal transition mainly depends on the spatio-temporal domain-based localization of ictal onset. However, the visual analysis of SEEG in neocortical seizures is extremely difficult due to rapid propagation of ictal discharges facilitated by dense intralobar and interlobar connectivity (8).

The major challenge in localizing the EZ from SEEG recording is the precise detection of ictal onset patterns (frequency change) and spatio-temporal separation of seizure onset zone from the propagation. Seizure onset in SEEG recordings is characterized by any of the following patterns (1) low-voltage fast activity (LVFA), (2) preictal spiking with rhythmic spikes of low frequency followed by LVFA, (3) burst of polyspikes of high frequency and amplitude followed by LVFA, (4) slow wave or baseline shift followed by LVFA, (5) rhythmic spikes or spike-waves, at low frequency and with high amplitude, and (6) theta/alpha sharp activity with progressive increasing amplitude (9, 10). Among these patterns, SEEG signature of LVFA was found to be largely observed (9) and the other onset patterns like burst-suppression and delta brush patterns were very rare and frequency adjustments in detector was required to detect such seizure onset from SEEG (9–11). Seizure onset from SEEG can be detected when there was abrupt change in energy ratio (ratio of emergence of fast oscillation replacing the slow oscillations) of the signal. A cumulative sum algorithm' or ‘CUSUM’ can be used to detect seizure onset zone by performing a test on the mean of baseline energy calculated from about few minutes of SEEG prior to ictal onset provided EEG seizures are absent (11–13).

Computational tools including Epileptogenicity Index (EI) (11), Epileptogenicity map (14, 15), Labview tool (16), Connectivity Epileptogenicity Index (cEI) (17), and Graph theoretical and machine learning-based approaches (18, 19) were developed to localize the EZ. Among these methods, EI quantifies the epileptogenicity in patient brain using spectral (appearance of abrupt frequency change) and temporal (delay of involvement of brain structure in seizure with respect to seizure onset) properties of SEEG. EI indexes the brain circuit between values “1” (more epileptic) and “0” (less epileptic). The brain area with EI value above 0.3 is recommended as an epileptic brain circuit (20). Though the EI has been defined and invented for mapping fast activities from different sources of seizure onset, this method worked well in mesial temporal seizures where the signal propagation was slow whereas it showed low accuracy (for localizing EZ) in neocortical seizures where the seizure propagation was rapid.

Epilepsy is a network disease, identifying the potential network hubs that initiates epileptic activity is the primary aim of presurgical evaluation (20). Unlike mesial temporal epilepsy, in neocortical epilepsy, the seizures propagate rapidly to the adjacent and anatomically connected areas of the brain that may be facilitated by the cytoarchitecture and short intralobar, interlobar, and interhemispheric connections in the cortex (8). In the literature, EI has been applied in studies of frontal lobe epilepsies (21), focal epilepsy with involvement of ictal discharges in thalamus and basal ganglia (22), heterotopic cortex (23), insular epilepsies (24), and posterior cortex epilepsies (25) with varying thresholds (EI > or = 0.6 or 0.3) as estimated values for localizing the EZ. However, these studies were not suggesting a definite threshold (index) for precise localization of EZ, especially in neocortical epilepsy. This could be due to rapid propagation of ictal discharges in the anatomically connected cortical areas. In these cases, the temporal based epileptogenicity quantification alone may not be enough to localize the EZ. In this study, we postulate that adding spatial parameters along with the EI equation can help to overcome such scenarios.

In the current study, we utilized the time of involvement of brain structures and Euclidean distance between brain structures, along with the energy ratio to localize the epileptogenic zone. Also, we proposed a modified method of EI called Epileptogenicity Rank (ER) to quantify epileptogenicity, especially in neocortical epilepsy. Two important questions we attempted to address in this study were (1) how to quantify epileptogenicity in neocortical epilepsy and derive a parameter to differentiate seizure onset from seizure propagation network, and (2) To find the optimum ER threshold value that accurately localized the EZ in mesial temporal and neocortical epilepsy. This modified method was implemented in MATLAB.

## Methods

### Patient Selection and Data Collection

In our study, SEEG recordings from 33 patients (including 23 males and 10 females with a mean age of 24.9 years) evaluated during 2015–2018 were included. Out of 33 patients, 27 of them were seizure-free (Engel 1 a-d) and 6 were non-seizure-free. For ER threshold estimation, our inclusion criteria were (1) the patients who underwent intracranial EEG evaluation with depth electrodes (Stereo EEG), (2) availability of post-op MRI, and (3) seizure-free/free of disabling seizures (Engel 1 a-d) till the last follow-up (26). The average outcome follow-up was 40.9 months. 6 non-seizure-free patients were analyzed to study how ER localizes EZ in epilepsy surgery failure patients with Engel 2 or 3 outcome. The epilepsy surgery outcome was collected from medical records of the post-OP clinic. The pre-surgical evaluation findings for all patients are given in Supplementary Table 1. The study was approved by the institutional review board.

SEEG electrodes were implanted in these patients using ROSA (Robotized Stereotactic Assistant) method (27). Patients were implanted with PMT (PMT Corp. USA) intracranial SEEG electrodes and the acquisition was done at 256 or 1,024 Hz using Nicolate/Natus 128 channel amplifier. After intracranial evaluation, patients underwent tailored resections of the identified epileptogenic zone. Within 12 months duration, a postoperative volumetric 3T MRI of the brain was acquired (using Siemens Magnetom Verio or GE Discovery MR 750 W) for each patient and these images were co-registered to volumetric CT images of the brain with intracranial electrodes to visualize the cortex recorded by the SEEG contacts and locate the electrode contacts within the resection cavity (28, 29). Image acquisition parameters were described in (30).

### Localizing Electrode Location From Post Implantation CT Images

**Figure 1:**
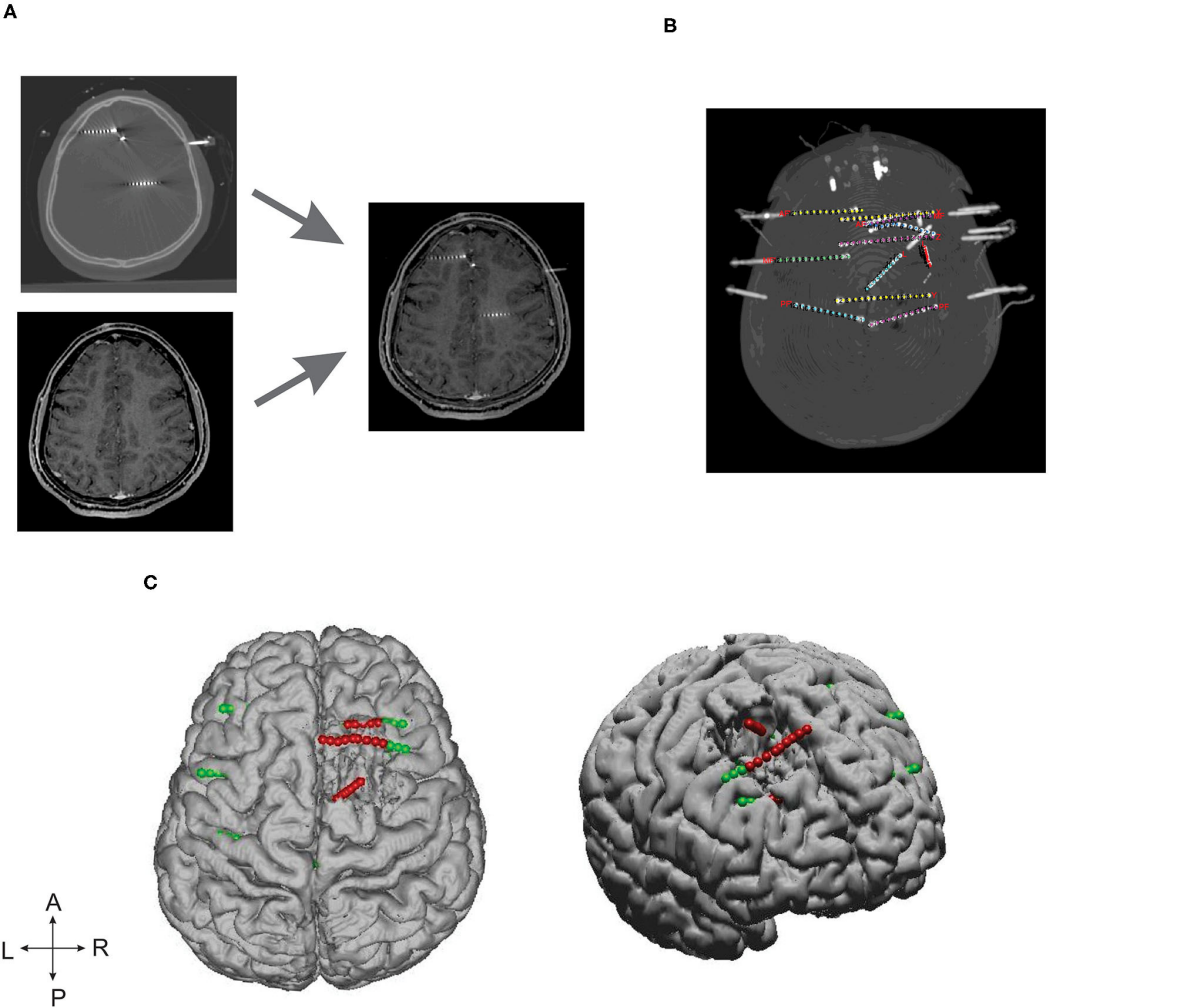
SEEG electrode implantation and post-processing of images. **(A)** Depicts the post-SEEG electrode implantation CT coregistered on post resection MRI. **(B)** Segmented and labeled SEEG electrodes. **(C)** Coregistered SEEG electrode contacts and the brain resection cavity. Axial (left) and lateral (right) patient brain model reconstructed from post-op MRI. Green dots represent the implanted SEEG electrodes, and red dots represent the electrode contacts identified within the resection cavity.

Advanced image processing techniques were required to localize SEEG electrodes from CT images, thanks to GARDEL, a computational tool for automatic segmentation and labeling of SEEG electrode contacts (29). In our study, we used GARDEL for (1) to coregister and localize SEEG electrodes in post-op MRI and (2) to export 3D coordinates of SEEG electrode contacts. Each electrode was manually assessed to identify SEEG contacts within the brain resection cavity (Figure 1).

### Implementation of Epileptogenicity Rank

ER calculates epileptogenicity as a function of energy ratio, time of involvement of brain structures, and the Euclidean distance from the initial seizure onset. ER requires two parameters to localize the EZ: (1) SEEG and (2) the 3D coordinates of SEEG electrode contacts. The EZ localization was partially automated by converting the time-series data to the frequency spectrum and applied a threshold over the mean activity to detect the seizure onset. Page and Hinkely's algorithm was implemented for seizure onset detection (12, 13). The proposed new method was implemented in a graphical interface for easy usability and made available at https://github.com/Brain-Mapping/EPI-rank.

#### Calculation of Energy Spectral Density (es) and Energy Ratio (er) From the SEEG

Epileptogenic zone localization was implemented in three steps: (1) calculation of energy spectral density from SEEG, (2) the optimal detection of seizure onset, and (3) calculation of epileptogenicity rank (ER). *S(t)* was a mono channel SEEG recorded during a seizure which consisted of interictal, pre-ictal, ictal, and post-ictal states. The computation of spectral density *(E)* of *S(t)* was described in (11). SEEG analysis was performed on a bipolar derivative montage (subtraction of consecutive channels).

#### Calculation of Epileptogenicity Rank (ER)

ER was calculated as the normalized values of the product of spatio-temporal parameter and energy of the signal. The spatial parameter was added along with the existing temporal domain based index calculation (EI) to bring the new epileptogenicity rank (ER). We set the range of ER from 1 to 10, “ER = 1” being highly epileptogenic and normal brain region ranked as “ER = 10.” ER of an SEEG was given by,


(1) $$\begin{array}{l} ER_{i}=\;\left[\frac{1}{(t_{i}-t_{0})\;+\;\alpha }\left(\frac{E_{\beta }+E_{\gamma }}{E_{\theta }+E_{\alpha }\;}\right)\right]+\\ \left[\;\frac{1}{d_{i}\;+\;\alpha }\left(\frac{E_{\beta }+E_{\gamma }}{E_{\theta }+E_{\alpha }\;}\right)\right] \end{array}$$

(2) $$\begin{array}{r} d_{i}=\;\sqrt{{\left(x-x_{i}\right)}^{2}+\;{\left(y-y_{i}\right)}^{2}+\;{\left(z-z_{i}\right)}^{2}} \end{array}$$

where *S*_*i*_ denotes the SEEG recorded from electrode contact *i* and 3D coordinate of *i* was given by *x*_*i*_*,y*_*i*_*,z*_*i*_*. t*_*i*_ denotes the time at which the frequency change was detected for electrode contact *i*. “*t*_*o*_” denotes the very first detection of frequency changes in “*t*_*i*_.” *E*_θ_*, E*_α_*, E*_β_, and *E*_γ_ denotes frequency bands theta, alpha, beta, and gamma, respectively (31); *d*_*i*_ denotes the Euclidean distance between the electrode contact ‘*i' (x*_*i*_*,y*_*i*_*,z*_*i*_*)* and the onset electrode contact (x,y,z); α denotes the constant value to avoid division by zero for the first frequency change detection.

### Estimating Optimum Epileptogenicity Rank for Mesial-Temporal and Neocortical Epilepsy

To estimate optimum ER, for each patient, we identified the SEEG electrode contacts within the brain resection cavity from their post-op MRIs (Figure 1). For each seizure, the maximum value of ER to localize the EZ (ER_max_) was estimated by increasing the value of ER from ER = 1 to “n;” the value of “n” was considered as maximum (ER_max_) when further increasing of ER resulted in localization of EZ outside the resection cavity. The optimum value for the ER was estimated in this study by including the data only from seizure-free patients. The variability in ER_max_ was calculated as mean ± standard deviation with a 95% confidence interval, and the optimum threshold for ER was estimated separately for mesial temporal and neocortical epilepsy by computing the ROC. For each patient, all stereotyped electro clinical seizures were included in the analysis. Patients with slow onset patterns were excluded from the analysis (P 4, 7, and 25 in Supplementary Table 1).

The surgical resection cavity in Engel I patients does include the epileptogenic zone, but also other brain tissue that needed to be resected to path the way to the “true” epileptogenic zone. This scenario is more obvious in mesial temporal lobe epilepsy where the standard epilepsy surgery (ATLAH: Anterior Temporal Lobectomy + Amygdalo-Hippocampectomy) was offered. In order to accommodate this exception, in our analysis, the seizure onset zone identified by epileptologist was considered as the gold standard for all patients with mesial temporal lobe epilepsy.

### Statistical Analysis

To estimate a threshold for EZ localization using ER, a receiver operating characteristic (ROC) curve was computed. ROC was plotted by estimating the sensitivity and specificity at different threshold ranges from ER = 1 to 10. McNemar's chi-square test was performed to assess the difference in EZ localization by EI and ER methods (32). The SEEG contacts were assigned to binary values (EZ localizations = 1 and others = 0) to compute the chi-square. To assess the quality of the methods, the percentage agreement between the EZ localization and the SEEG contacts identified within the resection cavity was also calculated. The percentage agreement was calculated as (no. of correct localization) / (no. of correct localization + no. of incorrect localization).

## Results

Of 27 patients included in our study, nine were diagnosed and treated for mesial temporal and 18 were diagnosed and treated for neocortical epilepsy and all were seizure-free till the last follow-up with a mean period of 40.9 months. An additional six non-seizure-free patients were also analyzed to study how ER localizes EZ in epilepsy surgery failure patients. Presurgical findings and patient details for all 33 patients were given in Supplementary Table 1.

### ER and EI Methods Localized the EZ Identically for Temporal Lobe Epilepsy and Differently in Neocortical Epilepsy Patients

**Figure 2:**
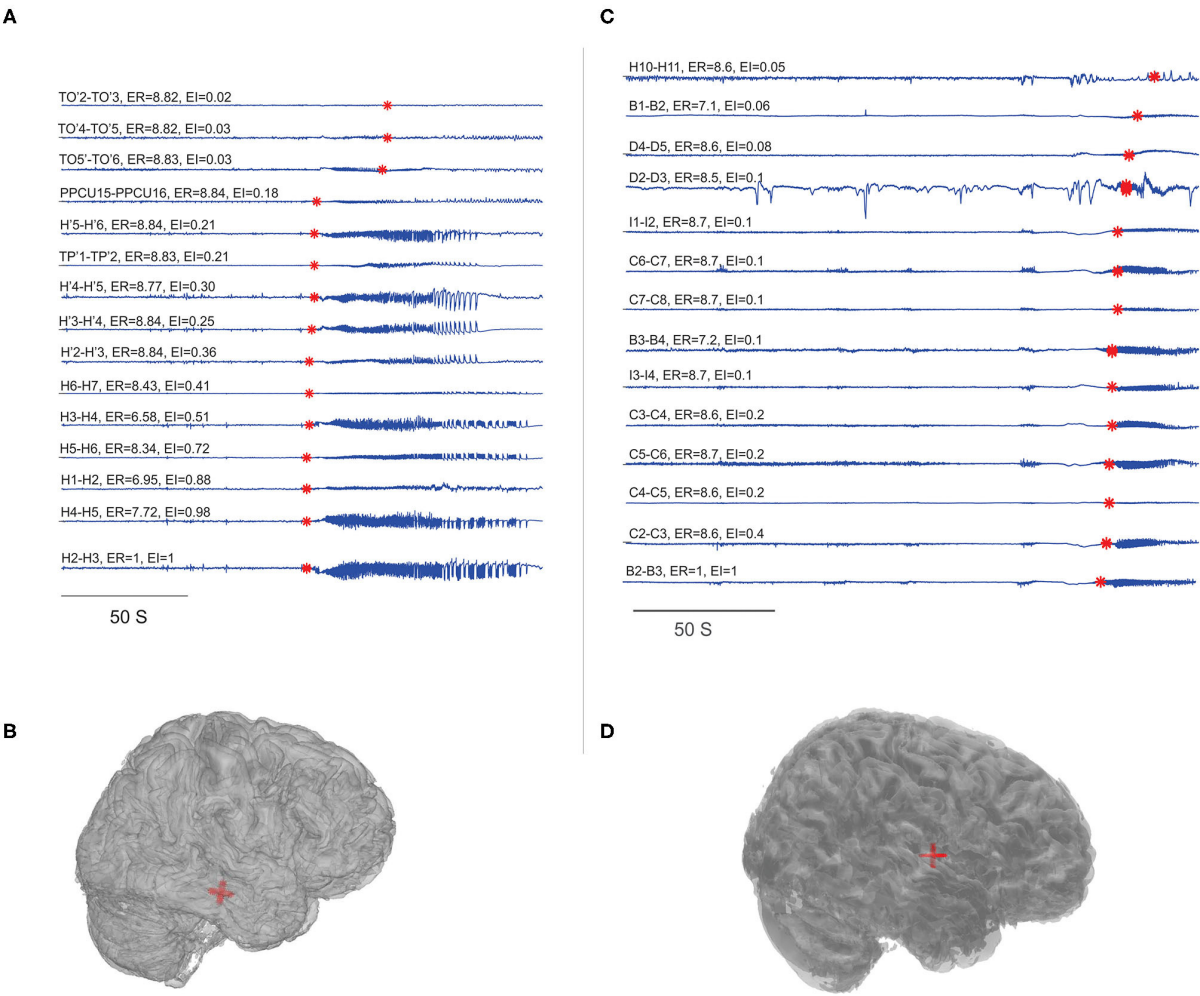
Localization of EZ in mesial temporal and neocortical epilepsy. EI and ER estimated for the patients P23 and P8. **(A,C)** SEEG during preictal to ictal transition, the red mark on each signal indicates the SEEG onset detection by the algorithm and the calculated ER and EI values were mentioned above the respective channels. **(B,D)** Were the patient brain model and the red shaded area indicated the electrode contacts with ER < 7.3. From bottom to top (in **A**), very first ictal discharge was detected in H 1-4 right anterior Hippocampus then spread to left anterior Hippocampus H' 1-4 then to TP' 1-2left amygdala and then to TO'2-TO'6 left temporo-occipital regions. From bottom to top (in **C**), very first ictal discharge was detected in B 2-3 and C 2-3 mid insula then immediately spread to rest of the contacts implanted in the insula. See Supplementary Table 5 for SEEG electrode names and abbreviations.

**Figure 3:**
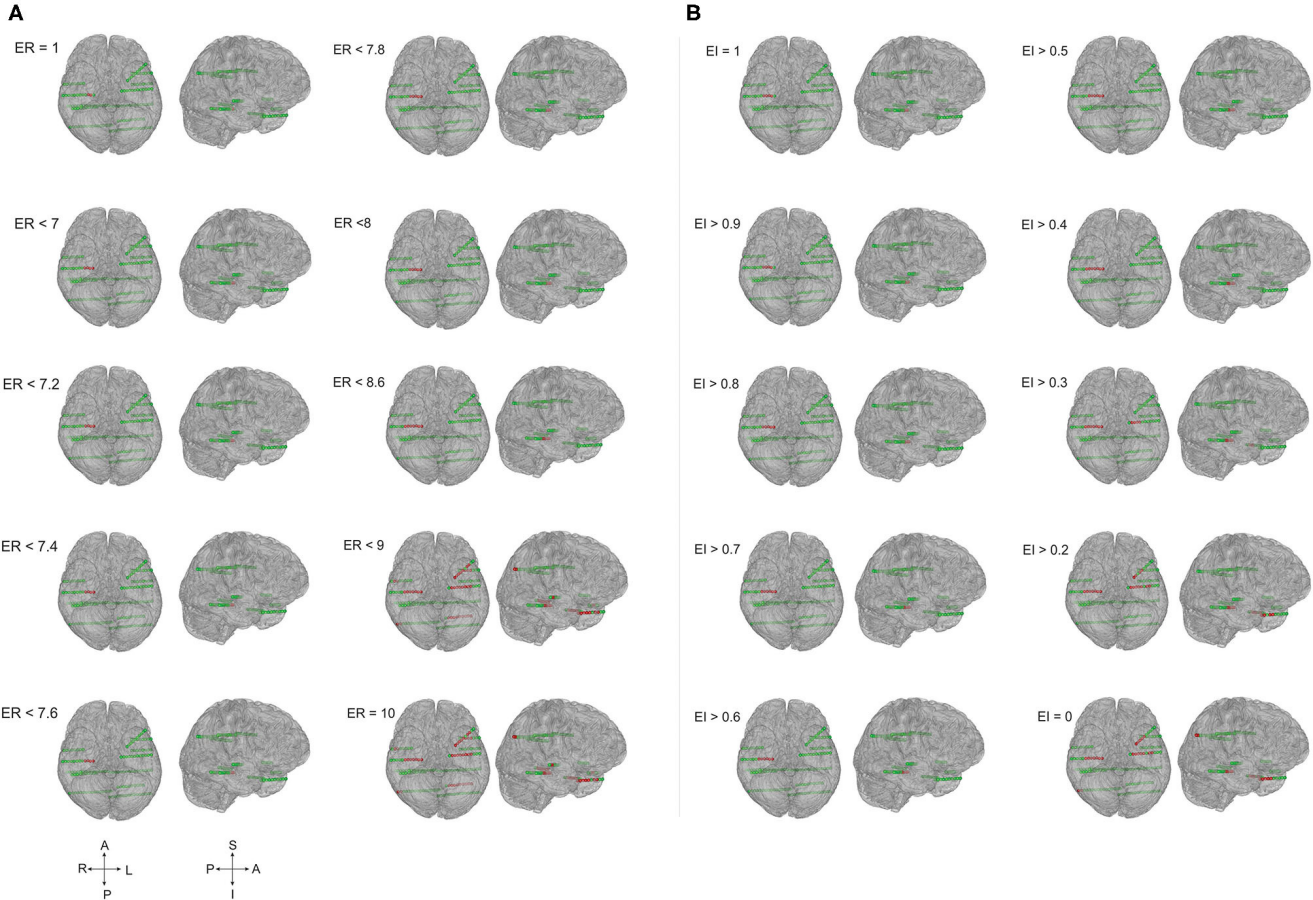
The ER and EI methods localized the EZ identically in temporal lobe epilepsy. Schematic of the patient brain (P23 in Supplementary Tables 1, 3) and the SEEG electrode contacts with color indicates, green = normal and red = localized as epileptic. **(A)** EZ localized by computing epileptogenicity using ER method and **(B)**. Epileptogenicity calculated using EI method. ER and EI were calculated for multiple thresholds to analyze the seizure onset zone and propagation. Comparing the localization of EZ by ER and EI; the value of ER varied from 1 to 10 whereas the EI varied from 1 to 0. The ER and EI methods localized the EZ identically in **(A,B)** (temporal lobe epilepsy) with the *p*-value = 0.39 in McNemar's chi-square test.

**Figure 4:**
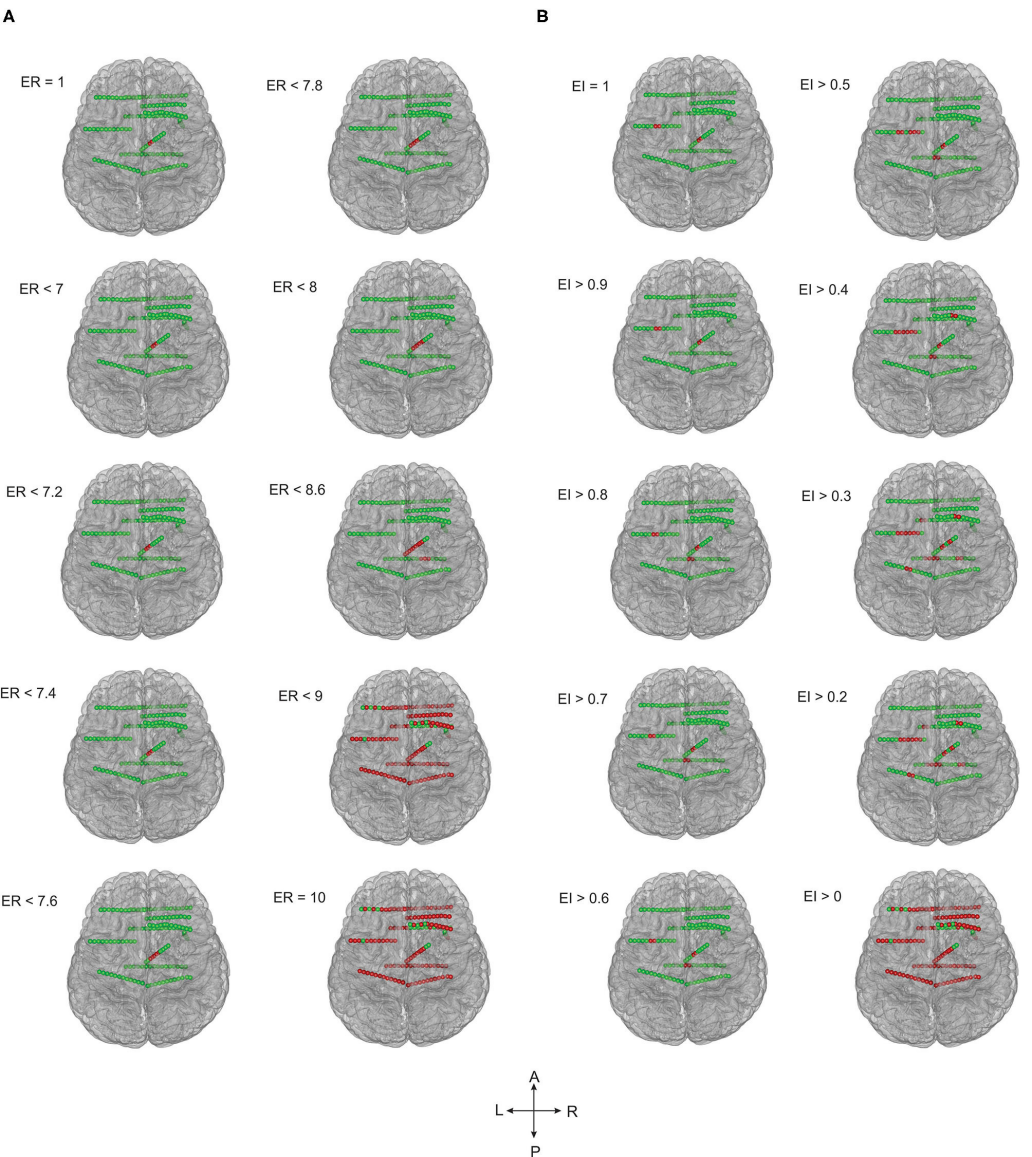
EZ localization by ER and EI methods contrasted significantly in frontal lobe epilepsy. The patient brain model (P1 of Supplementary Table 1) and the SEEG electrode contacts were showed (green = normal; red = localized within EZ). Epileptogenicity calculated by ER **(A)** and EI **(B)** methods. ER and EI were calculated for various thresholds (ER varied from 1 to 10 whereas the EI varied from 1 to 0) to localize the seizure onset from the propagation network. The EZ localization using ER and EI differed significantly **(A,B)** in neocortical epilepsy with the *p*-value = 0.01 in McNemar's chi-square test.

In this study, we compared the EZ localization of ER and EI based methods in mesial temporal and neocortical epilepsy. The seizure onset and the propagation network were computed by setting the threshold, EI > 0.3 and EI > 0.6 for both the group of patients, and in ER based method, the computed optimum ER values were used, ER < 7.1 for neocortical epilepsy and ER < 7.3 for the temporal lobe epilepsy group (see section Spatiotemporal Quantification of Epileptogenicity Has Helped to Map the Spatial Extent of EZ in Mesial Temporal and Neocortical Epilepsy). We observed that ER and EI methods localized the EZ identically in mesial temporal epilepsy with the *p*-value = 0.39. In neocortical epilepsy patients, EZ detection by the two methods significantly differed with *p*-value = 0.01 using McNemar's chi-square test (see Figures 2–4 and Supplementary Tables 2, 3).

In temporal lobe epilepsy, the percentage agreement between the EZ localized and SEEG contacts identified within the resection cavity was found to be 93.06% in ER method and the percentage agreement reduced to 65.60% in EI method. The percentage agreement in neocortical epilepsy was found to be 95.7% in ER, and the percentage agreement reduced to 51.3% in the EI method. From our analysis, we found that the ER (spatio-temporal) based localization could better differentiate seizure onset zone from propagation when compared to temporal domain based EI method (see Figures 3, 4 and Supplementary Tables 2, 3).

### The Spatial Extent of Epileptogenic Network and Underlying Etiologies

**Figure 5:**
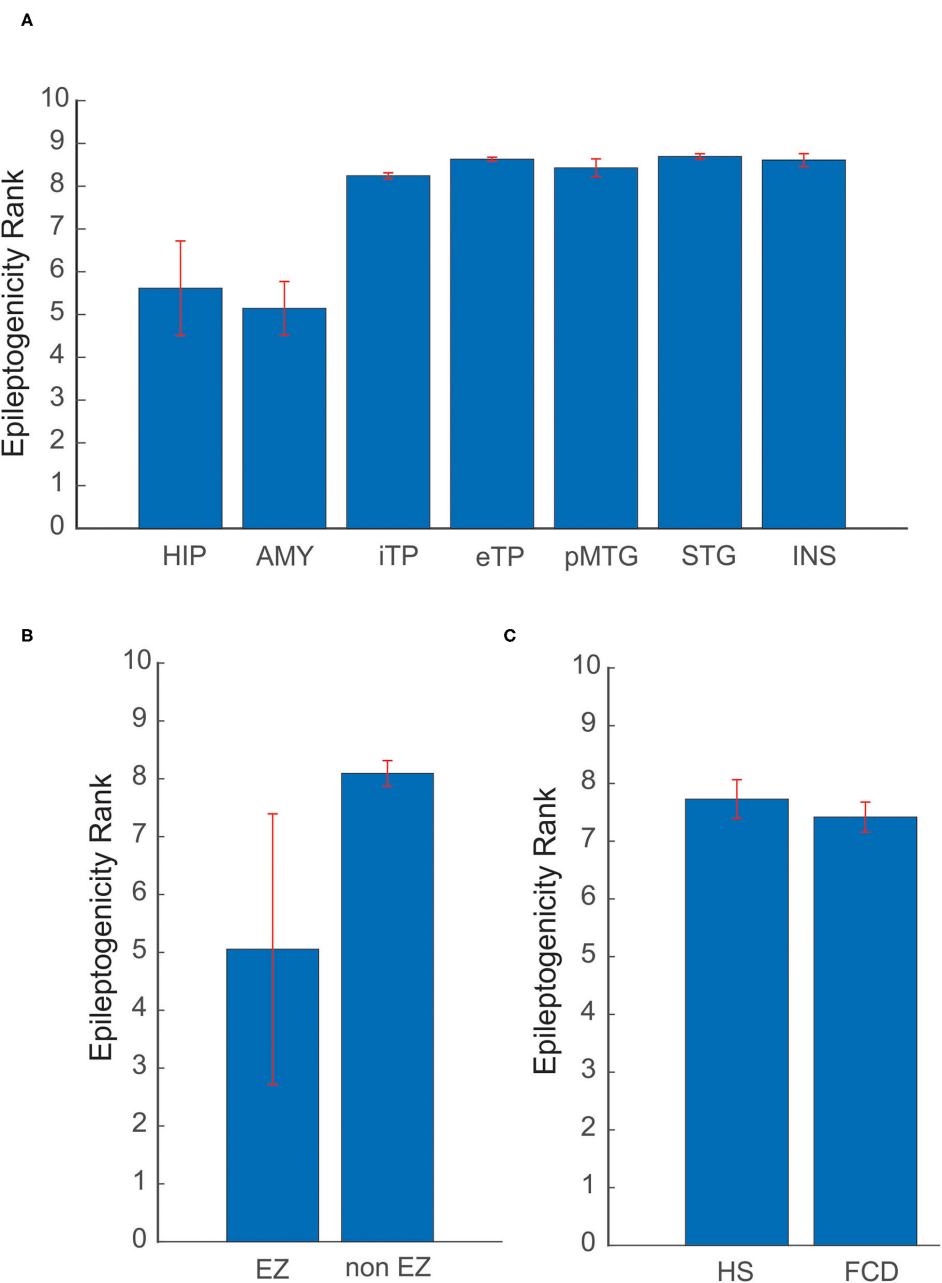
Variability of ER in different etiologies and brain structures. **(A)** Comparison of mean ER max between mesial and lateral structures in mesial temporal lobe epilepsy. **(B)** In neocortical epilepsy, ER max was compared for brain structures that come within EZ and non-EZ regions. **(C)** Localization utility of ER between Hippocampal Sclerosis (HS) and Focal Cortical Dysplasia (FCD). In **(A)** HIP, Hippocampus; AMY, Amygdale; iTP, Internal Temporal Pole; eTP, external Temporal Pole; pMTG, posterior part of the Middle Temporal Gyrus; STG, Superior Temporal Gyrus; INS, Insular cortex. The mean and standard deviation of ER max represents the spatial network of EZ in patients analyzed in this study.

The spatial extent of the epileptogenic network was studied in mesial temporal lobe and neocortical epilepsy patients. In the mesial temporal lobe epilepsy group, the ictal involvement was studied in mesial and lateral temporal structures and in neocortical epilepsy ictal involvement in EZ and non-EZ regions were considered. The mean and standard deviation of ER_max_ was estimated, in Hippocampus (5.61 ± 1.1), Amygdale (5.14 ± 0.62), Internal Temporal Pole (8.24 ± 0.06), external Temporal Pole (8.63± 0.04), posterior part of the Middle Temporal Gyrus (8.42 ± 0.2), Superior Temporal Gyrus (8.69 ± 0.06), and Insular cortex (8.61 ± 0.14). The mesial structures showed a significantly reduced ER_max_ when compared to lateral structures, indicating a high epileptogenicity for the mesial structures. In neocortical epilepsy, the brain structures that come within EZ showed significantly decreased ER_max_ of 5.05 ± 2.33, again suggesting high epileptogenicity when compared to the non-EZ regions (8.09 ± 0.22) (see Figures 5A,B).

Localization utility of ER between different etiologies, including Hippocampal Sclerosis (HS) and Focal Cortical Dysplasia (FCD), was also analyzed. The optimal ER (ER_max_) for HS was found to be 7.72 ± 0.33 and for FCD 7.41 ± 0.25. The mean ER_max_ for HS and FCD showed a difference of 0.31 in ER value (see Figure 5C). This difference may point to the underlying neuronal connectivity in different etiologies.

### Spatiotemporal Quantification of Epileptogenicity Has Helped to Map the Spatial Extent of EZ in Mesial Temporal and Neocortical Epilepsy

**Figure 6:**
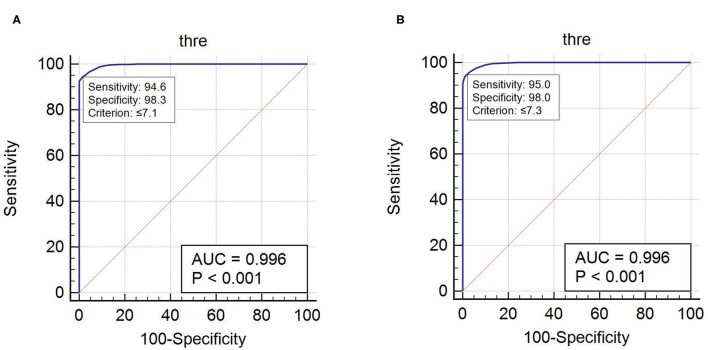
The optimum threshold for localizing EZ in ER method was calculated using ROC curve. **(A)** Optimum ER calculated for neocortical epilepsy. **(B)** Optimum ER calculated for mesial temporal epilepsy.

ER and EI based method localized EZ in patients with mesial-temporal lobe and neocortical epilepsy. The optimum threshold for detecting EZ in EI based method was set to 0.3 for both groups of patients for localizing EZ. The optimum threshold for localizing EZ based on the newly proposed method (ER) was estimated in this study using ER_max_ of all seizure free-patients (see Supplementary Tables 2, 3). We observed that ER < 7.1 was the optimum threshold to localize the EZ in neocortical epilepsy with a sensitivity of 94.6% and specificity of 98.3% and for mesial temporal lobe epilepsy the optimum ER threshold was estimated as ER < 7.3 with a sensitivity of 95% and specificity of 98% (Figure 6). These thresholds for ER gave top-left corner curves for both the ROC with the AUC (test accuracy) value of 0.996 (see Figure 6).

### ER Estimation in Non-Seizure-Free Patients Revealed the Spatial Organization of the Ictal Onset Zone Beyond Brain Resections

**Figure 7:**
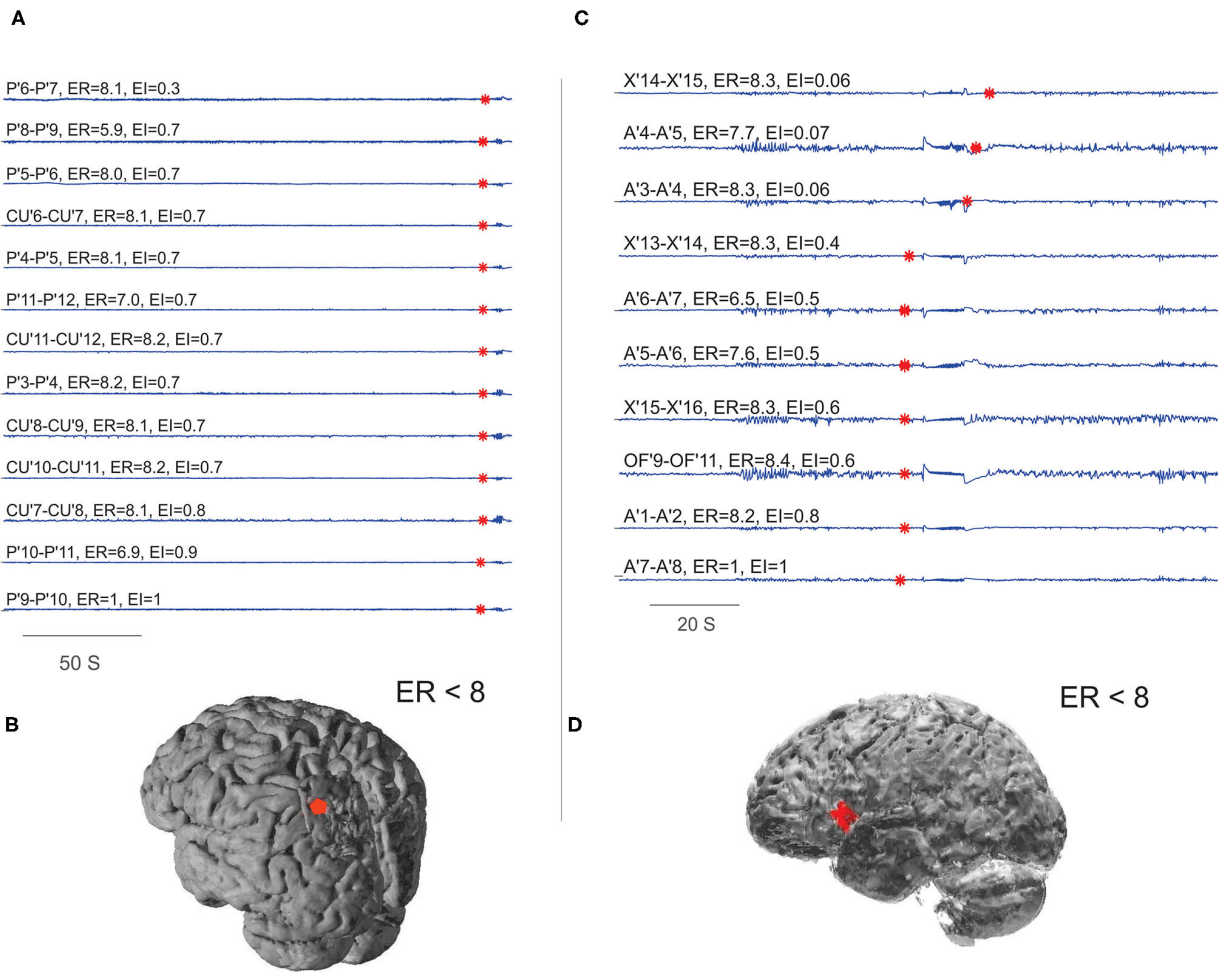
Localizing EZ in non-seizure-free patients. **(A,B)** Were the EZ localized for patient P28 and **(C,D)** for P29 in Supplementary Table 1. **(A,C)** Were the SEEG traces plotted during preictal to ictal transition; the red mark on each signal showed the detected frequency change. The estimated ER and EI values corresponding to brain areas were given above the respective traces. **(B,D)** Were schematic of the patient brain, and the red shaded area indicates the SEEG contacts within ER < 8. From bottom to top **(A)**, very first ictal discharge was detected in P' 9–11 left parietal cortex then immediately spread to CU' 7–11 cuneus. From bottom to top **(C)**, very first ictal discharge was detected in A'7–8 frontal operculum then it spread to OF'9–11 orbito frontal and X' 13–16 prefrontal cortex. See Supplementary Table 5 for SEEG electrode names and abbreviations.

With the availability of post-OP MRI, six post-SEEG epilepsy surgery failure patients were analyzed to study the spatial extent of EZ in non-seizure-free patients. In five out of six non-seizure-free patients, the ER method localized the EZ not only within the resection area but also to the areas adjacent to the borders of the resection cavity (see Figure 7 and Supplementary Table 4). The percentage agreement between localized EZ and the SEEG contacts within the resection cavity was found to be significantly less in non-seizure-free patients when compared to seizure-free patients (95.7% in seizure-free and 51.99% in non-seizure-free). The comparison between EI and ER methods in non-seizure-free patients showed less percentage agreement in EI (27.67%) compared to ER method (51.99%). The detailed statistical analysis on this comparison was limited by the low number of non-seizure-free patients in our study.

## Discussion

The main objective of this study was to formulate a scale (ER) to quantify epileptogenicity of brain structures in mesial temporal and neocortical epilepsy. We found that quantification of epileptogenicity by spatio-temporal method was more useful to differentiate ictal onset zone from the propagation when compared to temporal domain based EI method.

### Spatio-Temporal Based EZ Localization Method Better Differentiated the Seizure Onset Zone From Propagation Than the Temporal Domain Based Method

To differentiate sequence of events based on the temporal properties of signal (SEEG) alone is significantly complicated during the ictal period. The rapid propagation of ictal discharges was particularly evident in neocortical epilepsy, mainly facilitated by the cytoarchitecture and short intralobar, interlobar, and interhemispheric connections in the cortex (8, 18, 33–35). These fiber connections can be estimated using the tractography based on diffusion tensor imaging (36, 37). On the other hand, in the absence of fiber tractography distances, Euclidian distance between brain structures can be used to estimate the magnitude of cortico-cortical evoked responses (37–40). In our study, we used Euclidian distance between brain areas, time of involvement, and energy ratio to compute the ER.

We found a high percentage agreement in localizing EZ when anatomical distance parameters were introduced, along with temporal parameters in the calculation of epileptogenicity. In the comparative analysis of EI and ER method, we also observed that the temporal domain based estimations and localization often showed reduced EZ percentage agreement of 51.3% in neocortical epilepsy due to the fast propagation of signals in the ictal period. We also found a moderately high percentage agreement between localized EZ and SEEG electrode contacts within the brain resection cavity in ER when compared to EI. These findings suggest that temporal domain based calculation (EI) of epileptogenicity alone may not differentiate ictal onset from the propagation network, especially in neocortical epilepsy. With the spatio-temporal estimation of epileptogenicity as key strengths, this newly proposed “ER” method was helpful to localize EZ in neocortical epilepsy.

Estimating epileptogenicity threshold for localizing EZ is essential for different types of epilepsy. The optimum ER threshold for localizing EZ in mesial temporal and neocortical epilepsy was estimated and found to be ER < 7.1 for neocortical epilepsy and ER < 7.3 for mesial temporal epilepsy. This difference in the optimum threshold for localizing EZ may directly connect to the underlying differences in neuronal circuit and connectivity property that facilitates the seizure initiation and propagation in mesial temporal and neocortical epilepsy (41). Further, theoretical modeling and simulation of various SEEG onset patterns on the biophysical model of neuronal micro-circuit and computation of epileptogenicity can help to better understand the spatial organization of EZ (42–45).

### Studying the Spatial Organization of EZ in Different Etiologies

Drawing border for brain resection is still an open question in epilepsy surgery. The patients with large resections may have higher chance of more EZ area being resected. The standard for the spatial extent of EZ based on anatomical landmarks is also not always scientifically definable (18). The ER_max_ is different in different substrates/ brain regions as shown in our study, generalizing a threshold for localizing EZ and defining the borders of resection is difficult (18). To better understand these issues, we studied variation in epileptogenicity for localizing EZ in different etiologies like HS and FCD. The variation in ER value in these two groups of patients suggests that seizure initiation and propagation was considerably rapid in FCD (mainly neocortical epilepsy) with ER_max_7.41 ± 0.25 than in HS (mesial temporal lobe epilepsy) with ER_max_ 7.72 ± 0.33. This difference points to the underlying variation in neuronal connectivity across various etiologies and variation in mechanism of generation and propagation of ictal discharges. Many other factors, including the age of epilepsy onset, can also influence the variation in the spatial extent of epileptic brain circuits (11).

### EZ Localization in Non-Seizure-Free Patients Suggests That Spatial Organization of EZ Is Beyond Brain Resection

The post-SEEG epilepsy surgery outcome may be poor mainly because of three reasons: (1) the implantation hypothesis would have missed sampling the primary epileptic hub; (2) the resection of seizure onset zone in those patients was not adequate; and (3) generation of the secondary epileptogenic zone over time (46, 47). To understand some of these aspects, we studied EZ localization in a small population of non-seizure-free patients with the availability of post-OP MRI. The ER analysis of epilepsy surgery failure patients revealed interesting results on the spatial extent of the EZ. In five out of six post SEEG epilepsy surgery failure patients we analyzed, the EZ was localized to the brain structures not only within the resection zone but also to the resection borders. From our limited analysis, we suspect inadequate resection of the cortex to be one of the reasons for poor outcome. Further clinical validation and computational analysis is required on patients undergoing second SEEG evaluation after the first epilepsy surgery failure to prove these assumptions/hypotheses. Modeling and simulation of dynamics of seizure initiation and propagation in patient brain model can contribute to the current understanding of the spatial organization of EZ (48). The development of such detailed patient-specific epilepsy brain models should help better define brain resection border for epilepsy surgery.

### Limitations of the Study

The main assumption of quantification of EZ using ER was that the implanted SEEG electrodes always sample the seizure onset zone. Therefore, the current computational methods can fail when the SEEG electrode misses sampling the seizure onset zone. Other limitations of the studies are listed: (1) the optimal detection of frequency change in the ER method still relies on thresholding the detection parameter and (2) the inability to include all ictal onset patterns (especially the slow onset patterns) in the default detection method. Hence, more efficient algorithms need to be developed for the optimal detection of all ictal onset patterns in these non-stationary signals. The limitation with slow SEEG onset was recently studied with the graph theory and incorporated into a quantity, connectivity Epileptogenicity Index (cEI) (17). The comparison of ER and cEI was not performed in this study, since the current study involves patients with faster SEEG onsets. (3) ER thresholds estimated in this study were optimized for the current dataset, an independent test need to be conducted with other datasets. Also, a study with a larger number of patients is very essential to generalize the current results. “Fingerprint of the epileptogenic zone” is another interesting article which studied the fast gamma activity of SEEG contacts within the resection cavity for localization of EZ (18). They used machine learning algorithms to train the features extracted from the SEEG. Direct comparison between EZ fingerprint and ER may not be possible since we did not use advanced feature extraction and machine learning algorithms for the localization of EZ.

The spatial extent of brain resection for epilepsy surgery is still an open question. However, in clinical practice, the spatial extent of the brain resection is decided by integrating multiple modalities of presurgical evaluation. Computational localization of seizure onset will always complement visual analysis. The computational EZ localization has to be analyzed alongside with the semiology and the visual analysis of SEEG by expert epileptologist before finalizing the seizure onset zone for clinical applications.

### ER and Epileptic Hubs—A Future Study

Epilepsy is a network disease, identifying the potential network hub that initiates epileptic activity is the primary aim of presurgical evaluation. Our current study suggested that the spatio-temporal quantification of epileptogenicity can localize EZ from the local neuronal circuit activity recorded by the SEEG. Inclusion of these brain areas (EZ) involving primary epileptic hubs/ictal onset zone in the resection zone is the cornerstone for seizure freedom. Identification of primary epileptic hub in multilesional and non-lesional epilepsy cases can definitely improve the epilepsy surgery outcome. Targeting of epileptic hubs in these patients will open up an opportunity to use minimally invasive surgery like radiofrequency or laser ablation. Another important aspect of this study is on the generalizability of the findings of this new method. How does this threshold localize EZ in non-lesional vs large lesional patients? The research also needs to be extended to study the utility of ER with different underlying etiologies in larger number of patients.

## Conclusion

Quantifying epileptogenicity rank can help in localizing epileptic brain circuits for patient-specific surgical planning. Analyzing frequency components in SEEG with spatiotemporal information of rapid discharges between two brain structures was found to be a useful method to differentiate seizure onset from the propagation network. An important key to success for resective or minimally invasive epilepsy surgery depends on an optimal identification of the seizure onset zone and its propagation. We believe computational tools like ER can help to map EZ to a great extent and promise better seizure freedom. A detailed prospective study of ER based EZ localization on multilesional and non-lesional cases has to be conducted with a larger number of patients.

## Data Availability Statement

The original contributions generated for the study are included in the article/Supplementary Material, further inquiries can be directed to the corresponding author/s.

## Ethics Statement

This retrospective study involving human participants was reviewed and approved by Institutional review board, Amrita Institute of Medical Sciences, Kochi (IRB-AIMS-2020-195).

## Author Contributions

HP implemented the method and performed all the analysis in this manuscript. HP, SG, AP, and AK contributed to the design of the work, patient selection, and developing the manuscript. HP, SG, AP, SD, and AK contributed to the interpretation of the analysis. All authors contributed to the article and approved the submitted version.

## Funding

This study was supported by post-doctoral fellowship to HP from Amrita Institute of Medical Sciences, Kochi.

## Conflict of Interest

The authors declare that the research was conducted in the absence of any commercial or financial relationships that could be construed as a potential conflict of interest.

## Publisher's Note

All claims expressed in this article are solely those of the authors and do not necessarily represent those of their affiliated organizations, or those of the publisher, the editors and the reviewers. Any product that may be evaluated in this article, or claim that may be made by its manufacturer, is not guaranteed or endorsed by the publisher.
