# Supplementary material for: Quantification of Epileptogenic Network From Stereo EEG Recordings Using Epileptogenicity Ranking Method
Source: Front Neurol. 2021 Nov 3;12:738111. doi: 10.3389/fneur.2021.738111 (PMC8595106; doi:10.3389/fneur.2021.738111)
Supplement: Supplementary file 1 [file Data_Sheet_1.pdf]

Supplementary material

Table 1. Patient details. Engel scale: I Seizure free, II Rare disabling seizures, III Worthwhile improvement, IV No worthwhile improvement. The average post-op follow-up period was 40.9 months. ATLAH: Anterior Temporal Lobectomy + Amygdalo-Hippocampectomy. HS: Hippocampal Sclerosis. LVFA: Low-voltage fast activity. Type1 = Low-voltage fast activity (LVFA), Type 2 = Preictal spiking with rhythmic spikes of low frequency followed by LVFA, Type3 = Burst of polyspikes of high frequency and amplitude followed by LVFA, Type 4 = Slow wave or baseline shift followed by LVFA, Type 5 = Rhythmic spikes or spike-waves, at low frequency and with high amplitude and Type 6 = Theta/alpha sharp activity with progressive increasing amplitude.

Patients	Age	MRI	Epilepsy type	SEEG onset type	Type of Surgery	Pathology	Seizure freedom in Engel scale	Follow up in months
P1	15	Normal	Frontal	Type 3	Neocortical resection	Normal tissue	1a	37.4
P2	18	Normal	Frontal	Type 1	Neocortical resection	FCD 2a	1a	48.5
P3	27	Normal	Parietal	Type 3	Neocortical resection	No evidence	1c	38.6
P4	6	Mild atrophy of bilateral frontal lobes.	Frontal	Type 2	Neocortical resection	Reactive gliosis	1b	16.1
P5	43	Normal	Frontal	Type 3	Neocortical resection	Reactive gliosis	1b	28.4

P6	17	Gliosis involving the left superior parietal lobule and posterior temporal and basifrontal lobe.	Frontal	Type 1	Neocortical resection	Reactive gliosis	1d	39.1
P7	28	Normal	Frontal	Type 4	Neocortical resection	Inconclusive	1a	22.2
P8	11	Subtle volume loss in distal body and tail of left Hippocampus.	Frontal	Type 1	Neocortical resection	FCD 2b	1a	47.8
P9	47	Normal	Parietal	Type 3	Neocortical resection	Reactive gliosis	1d	37.6
P10	35	Subtle loss of digitation without significant volume loss of left hippocampus.	Frontal	Type 3	Neocortical resection	FCD 2b	1a	44.4
P11	26	Normal	Frontal	Type 1	Neocortical resection	Inconclusive	1a	32.9
P12	12	Mild HS	Frontal	Type 3	Neocortical resection	Reactive gliosis	1a	44.6
P13	27	Gliotic changes noted in right frontoparietal region	Frontal	Type 3	-	Reactive gliosis	1d	37.5
P14	20	Normal	Frontal	Type 1	Neocortical resection	FCD 2a	1a	45.5
P15	31	Normal	Frontal	Type 1	Neocortical resection	FCD 2a	1a	77.2
P16	31	Normal	Frontal	Type 1	Neocortical resection	FCD 2a	1a	66
P17	12	Normal	Frontal	Type 3	Neocortical resection	Reactive gliosis	1d	56.5

P18	28	Normal	Frontal	Type 3	Neocortical resection	Reactive gliosis	1d	14.2
P19	16	Right amygdala and temporal pole thickening of cortex and blurring of grey white junction	Temporal	Type 1 and 3	ATLAH	Dysplasia-Amygdyla	1a	52.2
P20	32	Bilateral HS.	Temporal	Type 1 and 3	ATLAH	HS	1a	52.4
P21	33	Mild HS	Temporal	Type 3 and 5	ATLAH	Reactive gliosis, Hippo neuronal loss	1a	32.7
P22	38	HS	Temporal	Type 1	ATLAH	MTS 1a	1a	14.2
P23	25	Normal	Temporal	Type 3	ATLAH	HS	1a	41.8
P24	22	Mild HS	Temporal	Type 1	ATLAH	HS	1a	34.3
P25	36	Right Mesial temporal sclerosis	Temporal	Type 5	ATLAH	Reactive gliosis, HS	1a	40.4

P26	25	HS	Temporal	Type 3	ATLAH	HS 1	1a	37.2
P27	31	Normal	Temporal	Type 3	ATLAH	FCD 2a, HS	1a	66.9
**Failure patients**								
P28	25	Normal	Parietal	Type 1	Neocortical resection	Reactive Gliosis with Possibility of Focal Cortical Dyslamination-Blumke's Type I	3	19.4
P29	27	Normal	Frontal	Type 3	Neocortical resection	Reactive gliosis	3	12
P30	15	Mild flattening of the left hippocampal gyrus. Relaxometry values are almost similar on both sides.	Parietal	Type 3	Neocortical resection	Normal tissue	3	7
P31	9	Normal	Frontal	Type 3	Neocortical resection	FCD-Type IIA	2	32.6
P32	33	Ulegyria & gliosis – left parietal lobe	Parietal	Type 3	Neocortical resection	Reactive Gliosis	3	35.8

P33	23	Normal	Frontal	Type 1	Neocortical resection	FCD-Type IIA	2	19.9

Table 2. ER and EI estimations of neocortical epilepsy patients

Patient No.	SEEG contacts identified within resection cavity	SOZ defined by the epileptologist	No. of seizures analyzed	EZ localized by Epileptogenicity Rank	EZ localized by Epileptogenicity Index (EI>0.3)	Optimized ER for brain resections	Percentage agreement between ER and resection cavity (ER < 7.1)	Percentage agreement between EI and resection cavity (EI > 0.3)	Percentage agreement between EI and resection cavity (EI > 0.6)
P1	L4-12<br>AF3-12	L4-7<br>AF 5-10	7	L3-10, AF4-11	L2-7, MF'2-8, PF'2-7, PF 1-9, AF 3-11, MF 6-7, Y12-13, AF'4-5, AF'9-11	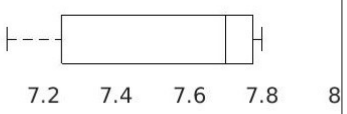	75	13.18	2
P2	PF' 7-12<br>F' 6-9	PF'6-12	12	PF'5-12	PF'4-12, F'3-10, Z'7-10, Y'5-13, P'1-7, PPCU'6-7, F'2-3, Y'12-16, Y'8-9	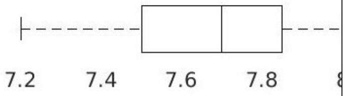	70.83	22.92	11.74
P3	SSMA 1-8	SSMA 4-7	3	SSMA4-8	SSMA2-8	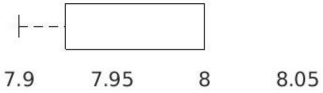	100	100	47.22
P5	A' 2-8	A'4-8	10	A'2-8	A'1-8,, B'4-6, X'6-8, Y'1-4, C'7-8	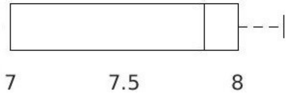	93.75	32.48	30.71
P6	D' 1-8<br>G'1-8	D' 2-4	8	D'2-7, G'3-6	D'1-7, B'1-2, E'3-8, H'8-10, G'4-6, P'4-5	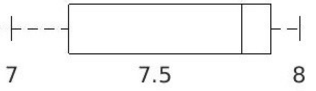	100	64.88	34.23

P8	I 1-8<br>B 1-8	I 6-7<br>B 1-8	2	I1-3, B1-4	I1-6, TP1-3, C7-8, C2-3, D1-2,B2-3,B6-7	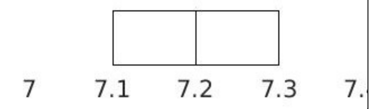	100	70.83	33.33
P9	PP’ 1-8<br>PPCU'9-12	PP'3-7	2	PP' 1-7	PP' 1-3, PP' 7-8, TO’9-10, PPCU’ 7-9, H'8-9	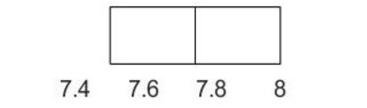	100	58.33	45.45
P10	A' 5-8	A’ 5-8	5	A'4-8	A'3-8, B'1-2, OF7-10, Y'9-13, OF'7-9, P'1-2, C'3-4	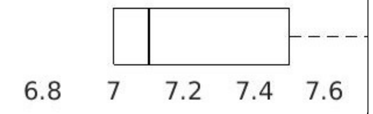	100	37.95	30.21
P11	MF 4-10	MF 6-9	9	MF4-10	MF 3-10, PF 2-10, PR2 1-2, AR1 -2, Y 8-9, AR2 2-3	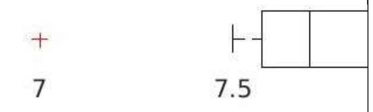	100	46.08	33.23
P12	Z1-3	Z 1-3	1	Z1-3	Z1-3	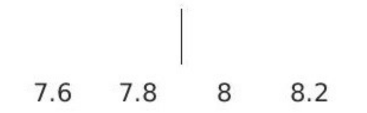	100	100	100
P13	AL 3-8<br>U 5-8<br>Pop 1-6<br>PF 9-10	U 6-8<br>AL 6-7<br>Pop7-8	6	Pop1-4, AL 5-10	Pop1-7, AL 1-7, PPCU4-5, PF9-10, Z5-7	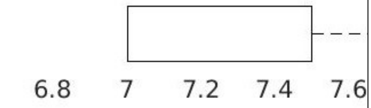	91.67	41.3	28.76
P14	A'3-8	A’ 5-7	2	A'3-8	-	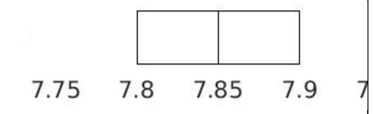	100	31.25	7.32
P15	FEF' 9-10<br>R' 1-3	FEF’ 9-10	2	FEF'8-10	FEF'4-10, PC'6-8, R'2-3		75	60	55

		R’1-3				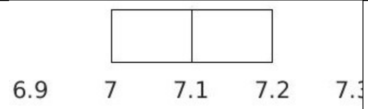			
P16	X' 1-10<br>FP' 1-10<br>AF' 1-10	Fp’1-3<br>X’ 1-2	4	FP'1-9	FP'1-8, FP2-3, X'1-2, H'1-3	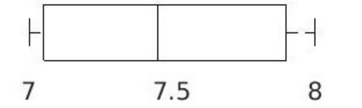	100	30	35.09
P17	Pcu 5-10,<br>P 1-10	P 2-3<br>Pcu 6-7	1	P2-5	P2-4	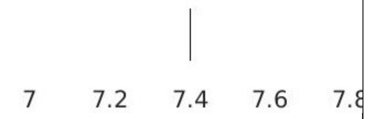	100	100	50
P18	SMA 7-8<br>F 3-10	SMA 7-8<br>F 1-10	2	F3-9	-	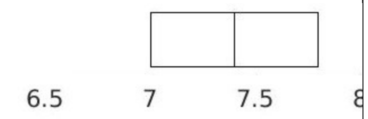	100	12.63	12.63
				Mean		**7.53**	**95.7**	**51.3**	**34.80**
				Std		**0.36**			

Table 3. ER and EI estimations of mesial temporal epilepsy patients

Patient No.	SEEG contacts identified within resection cavity	SOZ defined by the epilepto-logist	No. of seizures	EZ localized by Epileptogenicity Rank	EZ localized by Epileptogenicity Index (>0.3)	Optimized ER for brain resections	Percentage agreement between ER and resection cavity (ER < 7.3)	Percentage agreement between EI and resection cavity (EI > 0.3)	Percentage agreement between EI and resection cavity (EI > 0.6)
P19	H1-4<br>FP 3-10<br>OF 6-10	H1-3<br>FP5-10	9	H1-5, OF5-6	H1-4, A1-8, TO3-4, OF5-6, I5-6	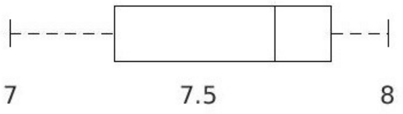	95.83	77.23	67.86
P20	H' 1-8<br>TP' 1-10	H’ 1-4<br>TP' 1-4	7	H'1-4, TP'1-4	H'1-6, TP'1-6, PH1-3, TP 5-7, PH'5-7	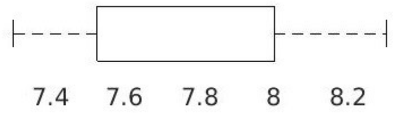	100	75.12	66.68
P21	TP' 1-8<br>H' 1-3	H’ 1-4<br>TP’ 1-7	9	H'1-6, TP'1-5	H'1-5, TP'1-6, D'1-6, B'2-3	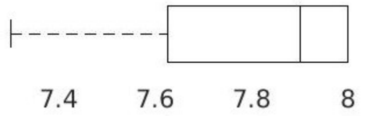	71.88	48.96	33.33
P22	TP 1-10<br>H 1-10	TP 1-5<br>H 1-4	3	TP1-4, H1-5	TP1-10, H2-6	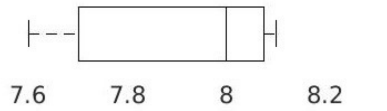	91.37	75	60
P23	H1-8	H 1-4<br>TP’1-4	7	H1-5, H'2-5, TP'2-4	H1-7, H'2-7, TP'2-8	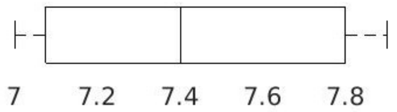	91	53.60	43.74

P24	TP 1-4, H1-9	H 1-2 TP 1-2	3	TP1-6, H1-4	H1-3, TP1-3, TP'9-10, TP9-10, H9-10	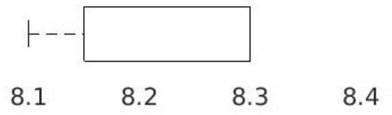	100	66.67	66.67
P25	TP 1-10 H 1-4	H 1-4	2	H1-4	H1-4	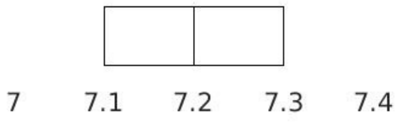	100	83.3	66.67
P26	H 1-6	H 1-4	6	H1-5	H1-4, H6-8, F1-8	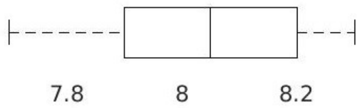	87.5	67.19	60.94
P27	E1-8 I 1-2 H 1-4	E1-3 H1-4	5	E1-6, H1-4	E1-5, A1-8, TO1-7, H1-6, I2-3	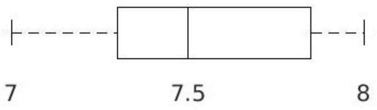	100	43.35	28.95
					Mean	**7.72**	**93.06**	**65.60**	**54.98**
					Std	**0.38**			

Table 4. ER and EI estimations of non-seizure free patients

Patient No.	Electrodes in resection cavity	Event No.	Epileptogenicity Rank (< 8)	Epileptogenicity Index (>0.3)	Percentage agreement between ER and resection cavity	Percentage agreement between EI and resection cavity
P28	P' 3-6<br>Cu' 3-7	10	P'5-12, CU'8-11	CU'1-14, P'1-12, PPCU'1-15, PR'2-3, O'1-2, O'5-9	13.33	26.86
P29	A' 2-8<br>MF' 1-7	3	A'4-8, MF'8-9	-	66.67	25
P30	PPCU 1-12<br>CU 1-10	7	PPCU'1-3, PPCu4-11, Cu7-11	-	50	41.59
P31	AR2' 1-4,<br>AR1'5-6,<br>PF'2-3	8	AR2'1-8, PR2'7-9, PR1'5-6, AR1'6-12	AR1'1-10, AR2'8-10, PR2'1-9, MF'2-3, PF'3-6	37.5	26.19
P32	PCU'5-10	3	PCU'2-10	PCU'4-9, PF' 1-10, PF7-8, Z'2-3, Z'9-10, TO3-5	100	32.91
P33	OF 1-10<br>FP 1-10<br>Y 7-12, TP 9-10	3	OF'8-10, TP 6-10, OF 6-10	TP7-10, FP'1-3, OF'1-10, OF2-3, OF9-10, H7-10, Y8-12	44.44	13.51
				mean	**51.99**	**27.67**

Table 5. Electrode name and abbreviations used in this study

Electrode name	Anatomical Name
H	Hippocampus
TP	Temporopolar
TO	Temporo-occipital
O	Occipital lobe
Cu	Cuneous
PPCU / Pop	Parieto-precuneus
P	Parietal
SM	Supramargical gyrus
G	Angular gyrus
SSMA	Secondary sensory motor area
Z	dorsal cingulate & supra marginal gyrus
Y	Anterior cingulate / transverse cingulate
A	Frontal operculam and anterior insula
B	Mid insula
C	Parietal operculam and posterior insula
D	Posterior inferior insula
E	Anterior inferior insula
I	Ventral insula / Longitudinal anterior insula
J	Longitudinal posterior insula
K	Longitudinal posterior insula
FP	Fronto-polar
OF	Orbito-frontal
X	Longitudinal anterior cingulate
F	Frontal
AF	Anterior frontal

MF	Mid frontal
PF	Posterior frontal (SMA)
AR	Anterior Rolandic
PR	Posterior Rolandic
U	Short gyrus posterior insula
L	Lesion
